# Supplementary material for: Perception towards cardiovascular diseases preventive practices among bank workers in Hossana town using the health belief model
Source: PLoS One. 2022 Feb 28;17(2):e0264112. doi: 10.1371/journal.pone.0264112 (PMC8884546; doi:10.1371/journal.pone.0264112)
Supplement: S1 Questionnaire — (DOCX) [file pone.0264112.s001.docx]

| **General Information** | Date _____________  Respondent ID ______________  Company/Bank name _______________ |
| --- | --- |

**Part I: Socio demographic information**

**Instruction: *for each of the following questions please circle the number of alternative(s) that fit for your response.***

| **No** | **Question** | **Response** | **Skip** |
| --- | --- | --- | --- |
| 101 | Age in years | _______years |  |
| 102 | Sex | 1. Male 2. Female |  |
| 102 | Educational level | 1. Only read and write 2. High school complete 3. Diploma 4. Degree 5. Masters |  |
| 103 | Marital status | 1. Single 2. Married 3. Divorced 4. Widowed |  |
| 104 | Religion | 1. Orthodox 2. Protestant 3. Muslim 4. Catholic 5. Others |  |
| 105 | Work experience in years | _______years |  |
| 106 | Current position/level in the bank | 1. Beginner 2. Officer position 3. Mid-level manager 4. Higher level manager |  |
| 107 | How many hours you work (per week) |  |  |
| 108 | Monthly income in ETB | ___________Birr |  |

**Part 2: Knowledge about CVD**

| **No** | **Question** | **Response** | **Skip** |
| --- | --- | --- | --- |
| 201 | Do you know about CVDs? | 1.Yes  2. No |  |
| 202 | If yes, which type of CVD do you know? List all you know | 1. Coronary heart disease (CHD) 2. Hypertension 3. Stroke 4. Heart failure 5. Rheumatic heart disease 6. congenital heart disease 7. Others (Specify______) |  |
| 203 | From where do you hear about CVD? (Source of information) (more than one response is possible) | 1. Mass media 2. Social media 3. Health workers 4. From work place 5. From friends 6. Others(Specify_____) |  |
| 204 | Do you know the risk factors for CVD? | 1.Yes  2. No |  |
| 205 | If yes, can you mention risk factors for CVD? (more than one response is possible) | 1. Lack of physical activity 2. Excessive alcohol intake 3. Tobacco Smoking 4. Reducing excess intake of salt and fat in daily diet 5. Staying long hours on office chairs 6. Passive smoking 7. Others(Specify_____) |  |
| 206 | Do you know how CVD can be prevented? | 1.Yes  2. No |  |
| 207 | If yes, can you mention some of the preventive measures of CVD? (more than one response is possible) | 1. Physical activity 2. Reduce alcohol intake 3. Quit tobacco Smoking 4. Avoidance of passive smoking 5. Consuming more fruit, nuts, seeds, vegetables 6. Reducing salt and fat from daily diet 7. Others(Specify_____) |  |

**Part 3: Behavioral risk factors**

| **No** | **Question** | **Response** | **Skip** |
| --- | --- | --- | --- |
| 301 | Have you ever smoked cigarette? | 1.Yes  2. No |  |
| 302 | Do you currently smoke cigarette? | 1.Yes  2. No |  |
| 303 | For how long did you smoke? | _________years |  |
| 304 | Have you exposed to passive smoking at home, workplace or other areas? | 1.Yes  2. No |  |
| 305 | Do you engage in any physical activity? | 1. Yes 2. No |  |
| 306 | If yes, in which type of physical exercise do you currently engaged? | 1. Moderate physical activity   (30 minutes for 5 days/week)   1. Vigorous physical activity   (15 minutes for 5 days/week)   1. Working in gym 3 or more days/week 2. Participate in football events 3 or more days/week 3. Others (Specify_______) |  |
| 307 | If no, why you didn’t engage in physical exercise? | 1. Don’t see the benefit of physical exercise 2. Being busy with office work 3. Other (Specify_______) |  |
| 308 | Do you take alcohol? | 1.Yes  2. No |  |
| 309 | If yes, how do you describe your alcohol intake? | 1.Somitimes  2. Occasionally  3. Regularly |  |
| 310 | How much unit alcohol do you take in average in a day? | _________ units  One unit = half pint of beer (5 % alcohol), 100 ml of wine (11 % alcohol), 25 ml of alcohol (40% alcohol) |  |
| 311 | Do you regularly eat fruits and vegetables in your daily meal? | 1. Yes 2. No |  |
| 312 | Do you regularly consume salt and fats in your daily meal? | 1. Yes 2. No |  |
| 313 | History of CVD | 1. Yes 2. No |  |
| 314 | Family history of CVD | 1. Yes 2. No |  |

**Part 4: Perception questions**

| **No.** | **Question** | **Response** | | | | |
| --- | --- | --- | --- | --- | --- | --- |
|  | **Perceived susceptibility** | **Strongly disagree** | **Disagree** | **Neutral** | **Agree** | **Strongly agree** |
| 401 | In my opinion, as I am working in office, I am susceptible to CVD | 1 | 2 | 3 | 4 | 5 |
| 402 | I feel I will get CVD sometimes during my life | 1 | 2 | 3 | 4 | 5 |
| 403 | It is likely that I will get CVD | 1 | 2 | 3 | 4 | 5 |
| 404 | I worry a lot about having CVDs | 1 | 2 | 3 | 4 | 5 |
|  |  |  |  |  |  |  |
|  | **Perceived severity** | **Strongly disagree** | **Disagree** | **Neutral** | **Agree** | **Strongly agree** |
| 405 | The thought of CVD scares me | 1 | 2 | 3 | 4 | 5 |
| 406 | When I think about CVD my heart beats faster | 1 | 2 | 3 | 4 | 5 |
| 407 | CVD would affect my work | 1 | 2 | 3 | 4 | 5 |
| 408 | I am afraid to think about CVD | 1 | 2 | 3 | 4 | 5 |
| 409 | If I got CVD, it would be more serious than other disease | 1 | 2 | 3 | 4 | 5 |
| 410 | CVD is not serious as other diseases | 1 | 2 | 3 | 4 | 5 |
| 411 | Death resulting from CVD is rare | 1 | 2 | 3 | 4 | 5 |
| ­­­­­­ |  |  |  |  |  |  |
|  | **Perceived benefits** | **Strongly disagree** | **Disagree** | **Neutral** | **Agree** | **Strongly agree** |
| 412 | When I do physical exercise, I am doing something to take care of myself | 1 | 2 | 3 | 4 | 5 |
| 413 | Regularly screening for CVD may help me to take care of myself | 1 | 2 | 3 | 4 | 5 |
| 414 | Regular physical exercise decreases the risk of CVD | 1 | 2 | 3 | 4 | 5 |
| 415 | If I do physical exercise regularly, I don’t worry much about CVD | 1 | 2 | 3 | 4 | 5 |
| 416 | If I do physical exercises regularly, I will decrease my chances of exposure to CVD | 1 | 2 | 3 | 4 | 5 |
| ­­­ |  |  |  |  |  |  |
|  | **Perceived barriers** | **Strongly disagree** | **Disagree** | **Neutral** | **Agree** | **Strongly agree** |
| 417 | Doing physical exercise is difficult to me | 1 | 2 | 3 | 4 | 5 |
| 418 | Doing physical exercise will take too much of my working hour | 1 | 2 | 3 | 4 | 5 |
| 419 | It’s hard to remember to do physical exercise regularly | 1 | 2 | 3 | 4 | 5 |
| 420 | Physical exercise is not necessary if I have regular screening for CVD | 1 | 2 | 3 | 4 | 5 |
| 421 | It’s difficult for me to do regular physical exercises | 1 | 2 | 3 | 4 | 5 |
| 422 | I have other problems more important than doing physical exercise | 1 | 2 | 3 | 4 | 5 |
| 423 | If I engage in regular physical exercise, it would lead me to worry about CVD | 1 | 2 | 3 | 4 | 5 |
| 424 | My friends/family would make fun of me if I engage in regular physical exercise | 1 | 2 | 3 | 4 | 5 |
| 425 | Doing regular physical exercise would require starting a new habit, which is difficult for me | 1 | 2 | 3 | 4 | 5 |
|  |  |  |  |  |  |  |
|  | **Perceived self-efficacy** | **Strongly disagree** | **Disagree** | **Neutral** | **Agree** | **Strongly agree** |
| 426 | I know how to do regular physical exercise to prevent CVD | 1 | 2 | 3 | 4 | 5 |
| 427 | I know when to do physical exercise if I have to do | 1 | 2 | 3 | 4 | 5 |
| 428 | I am confident I can do regular physical exercise | 1 | 2 | 3 | 4 | 5 |
| 429 | I think I can control CVD risk factors by myself | 1 | 2 | 3 | 4 | 5 |
| 430 | I can visit a health care provider to check my health status | 1 | 2 | 3 | 4 | 5 |
| 431 | I could feel any abnormalities in my health status | 1 | 2 | 3 | 4 | 5 |
|  |  |  |  |  |  |  |
|  | **Cues to action/Motivation** | **Strongly disagree** | **Disagree** | **Neutral** | **Agree** | **Strongly agree** |
| 432 | I have heard good things about physical exercise in preventing CVD | 1 | 2 | 3 | 4 | 5 |
| 433 | I have good support from my office to do regular physical exercise | 1 | 2 | 3 | 4 | 5 |
| 434 | I have seen friends engaged in regular physical exercise | 1 | 2 | 3 | 4 | 5 |
| 435 | I have seen my friends suffering from CVDs | 1 | 2 | 3 | 4 | 5 |

**Part 5: Perception about CVD preventive behaviours**

|  | **Perception about CVD preventive behaviours** | **Strongly disagree** | **Disagree** | **Neutral** | **Agree** | **Strongly agree** |
| --- | --- | --- | --- | --- | --- | --- |
| 501 | If I do regular physical exercise, I can reduce the risk of CVD | 1 | 2 | 3 | 4 | 5 |
| 502 | Refraining from tobacco smoking will reduce my risk of CVD | 1 | 2 | 3 | 4 | 5 |
| 503 | Regular taking of alcohol will increase the risk of CVD | 1 | 2 | 3 | 4 | 5 |
| 504 | Reducing salt and fat intake from daily diet will reduce the risk of CVD | 1 | 2 | 3 | 4 | 5 |
| 505 | Regular screening for possible CVD will reduce complications associated with CVD | 1 | 2 | 3 | 4 | 5 |

***Thank you for your cooperation to take part in the study!!!!***

Signature of the data collector____________________
