# Supplementary material for: Perception towards cardiovascular diseases preventive practices among bank workers in Hossana town using the health belief model
Source: PLoS One. 2022 Feb 28;17(2):e0264112. doi: 10.1371/journal.pone.0264112 (PMC8884546; doi:10.1371/journal.pone.0264112)
Supplement: S1 File — (DOCX) [file pone.0264112.s002.docx]

**
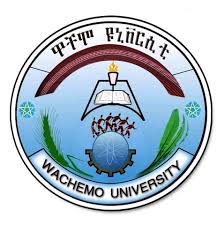
**

**WACHEMO UNIVERSITY**

**COLLEGE OF MDICINE AND HEALTH SCIENCE DEPARTMENT OF PUBLIC HEALTH**

**PERCEPTION OF BANK WORKERS TOWARDS CARDIOVASCULAR DISEASE PREVENTION IN HOSSANA TOWN: APPLICATION OF HEALTH BELIEF MODE**

**BY LEMLEM KIFLEYESUS**

**A RESEARCH PROPOSAL TO BE SUBMITTED TO WACHEMO UNIVERSITY COLLEGE OF MEDICINE AND HEALTH SCIENCE DEPARTMENT OF PUBLIC HEALTH IN PARTIAL FULFILLMENT OF THE REQUIREMENT FOR DEGREE OF MASTERS IN GENERAL PUBLIC HEALTH**

**FEBRUARY /2020**

**HOSSANA, SOUTH ETHIOPIA**

**PERCEPTION OF BANK WORKERS TOWARDS CARDIOVASCULAR DISEASE PREVENTION IN HOSSANA TOWN: APPLICATION OF HEALTH BELIEF MODEL**

**BY: LEMLEM KIFLEYESUS**

**ADVISORS: Dr. FELEKE DAYORE (PHD)**

**Mrs. AREGASH MECHA (MPH)**

**FEB, 2020**

**HOSSANA, SOUTH ETHIOPIA**

I, the undersigned, declare that this is my original work and has never been presented by another person in this or any other University and that all the source materials and references used for my proposal have been duly acknowledged.

Name:- Lemlem K/yesus

Signature: _______________________

Date of Submission: ______________

The proposal has been submitted for examination with my approval as a university advisor.

Name of advisors:-

1. Dr. Feleke. D. (PhD) -----------Main advisor

Signature: _____________________

Date: _________________________

1. Mrs. Aregash Mecha (MPH )---------Co-advisor

Signature: ______________________________

Date: ____________________________

# Acknowledgment

My heartfelt thanks go to Wachemo University college of Medicine and of health sciences, department of Public Health for giving me the academic opportunity to conduct my research thesis. My special thanks also goes to my advisors Dr. Feleke Doyore and Ms. Aregash Mecha for their unreserved support from the beginning of my research proposal development. I would like to extend my genuine thanks to Banks in Hossana town for giving me the necessary information to my study.

# Table of Contents

[Acknowledgment ii](#_Toc33171109)

[Table of Contents iii](#_Toc33171110)

[List of Tables v](#_Toc33171111)

[List of figures vi](#_Toc33171112)

[Acronyms and Abbreviations vii](#_Toc33171113)

[Summery viii](#_Toc33171114)

[Chapter one 9](#_Toc33171115)

[Introduction 9](#_Toc33171116)

[1.1 Background 9](#_Toc33171117)

[1.2 Statement of the problem 11](#_Toc33171118)

[1.3 Significance of the study 13](#_Toc33171119)

[Chapter two 14](#_Toc33171120)

[Literature review 14](#_Toc33171121)

[2.1 Prevalence of CVDs 14](#_Toc33171122)

[2.2 Risk factors of CVD 15](#_Toc33171123)

[Conceptual framework 19](#_Toc33171124)

[Chapter three 20](#_Toc33171125)

[Objective 20](#_Toc33171126)

[3.1 General objective 20](#_Toc33171127)

[3.2 Specific Objectives 20](#_Toc33171128)

[Chapter four 21](#_Toc33171129)

[Method and Material 21](#_Toc33171130)

[4.1 Study area and period 21](#_Toc33171131)

[4.2 Study design 21](#_Toc33171132)

[4.3 Population 21](#_Toc33171133)

*[4.3.1](#_Toc33171134)**[Source population](#_Toc33171134)* [21](#_Toc33171134)

*[4.3.2](#_Toc33171135)**[Study population](#_Toc33171135)* [21](#_Toc33171135)

[4.4 Eligibility criteria 21](#_Toc33171136)

*[4.4.1](#_Toc33171137)**[Inclusion criteria](#_Toc33171137)* [21](#_Toc33171137)

*[4.4.2](#_Toc33171138)**[Exclusion criteria](#_Toc33171138)* [22](#_Toc33171138)

[4.5 Sample size determination and sampling procedure 22](#_Toc33171139)

*[4.5.1](#_Toc33171140)**[Sample size](#_Toc33171140)* [22](#_Toc33171140)

*[4.5.2](#_Toc33171141)**[Sampling procedure](#_Toc33171141)* [23](#_Toc33171141)

[4.6 Data collection and measurement 24](#_Toc33171142)

*[4.6.1](#_Toc33171143)**[Data collection instrument](#_Toc33171143)* [24](#_Toc33171143)

*[4.6.2](#_Toc33171144)**[Pre testing of the questionner](#_Toc33171144)* [24](#_Toc33171144)

*[4.6.3](#_Toc33171145)**[Data collection methods and collectors](#_Toc33171145)* [25](#_Toc33171145)

[4.7 Study variables 25](#_Toc33171146)

[4.8 Data processing and analysis 25](#_Toc33171147)

[4.9 Data quality control 26](#_Toc33171148)

[4.10 Operational and definitions of terms and concepts 26](#_Toc33171149)

[4.11 Ethical consideration 27](#_Toc33171150)

[4.12 Dissemination plan 27](#_Toc33171151)

[Chapter five 28](#_Toc33171152)

[Work plan of the study 28](#_Toc33171153)

[Chapter six 29](#_Toc33171154)

[Budget Plan 29](#_Toc33171155)

[Refferences 30](#_Toc33171156)

[Annex: English version questionnaire 33](#_Toc33171157)

# List of Tables

[Table 1: Work plan to study the perception of bank workers in Hossana town, South Ethiopia 28](#_Toc29330633)

[Table 2: The total budget break dawn required to study the perception of bank workers in Hossana town, South Ethiopia 29](#_Toc29330634)

# List of figures

[Figure 1. Conceptual framework of the study by using health belief model 19](#_Toc29308056)

[Figure 2. Schematic presentation of sampling procedure to select study participants 23](#_Toc29308057)

# Acronyms and Abbreviations

| AHA | American Heart Association |
| --- | --- |
| CDC  CHD | Center for Communicable disease control  Coronary Heart Disease |
| CHF  CVD | Congestive Heart Failure  Cardiovascular Diseases |
| FMoH | Federal Ministry of Health |
| IHD  HBM | Ischemic Heart Disease  Health Belief Model |
| NCD | Non-Communicable Disease |
| SNNPR | South Nations and Nationalities Peoples Region |
| WHO | World Health Organization |
|  |  |
|  |  |
|  |  |

# Summery

**Background**: Cardiovascular disease (CVD) refers to a group of disorders of the heart and blood vessels. Cardiovascular diseases are responsible for a considerable proportion of the mortality and morbidity of the general population and they are becoming a public health problem in Ethiopia within a health care system that focus on communicable diseases. Bank workers are at increased risk of contracting as cardiovascular disease they spend most of their time in office chairs. Therefore, knowing bank worker’s perception to engage in cardiovascular disease preventive behavior has great importance.

**Objective**: is to assess the perception of Bank workers towards cardiovascular disease preventive behaviors and associated factors in Hossana town, South Ethiopia.

**Methods and materials**: A cross sectional study will be employed on a total of 258 study participants from February 11/2020 to April 20/2020. Data will be collected by using a self-administered structured questionnaire. Data will be coded, entered and analyzed by using SPSS version 20 software. Descriptive statistics, bivariate logistic regressions, general linear and multivariate analysis will be employed and the result will be described in words and figures. P-value less than 0.05 and 95% CI will be used to declare association between independent and the dependent variables.

**Work plan and budget**: The study will be expected to be conducted from February 11/2020 to April 20/2020 among bank workers of Hossana town with a total budget of **23,090** Ethiopian birr.

**Key words**: Cardiovascular disease, Perception, risk behaviors, health belief model, bank workers, Hossana town.

# Chapter one

# Introduction

## Background

Cardiovascular disease (CVD) refers to a group of disorders of the heart and blood vessels. These disorders include but are not limited to coronary heart disease (CHD), hypertension, cerebrovascular disease, peripheral artery disease, heart failure, rheumatic heart disease, and congenital heart disease(1).It is among the main causes of deaths around the world both in developed and developing countries, more people die annually from cardiovascular disease than from any other cause(2)

According to World Health Organization global health estimate of 2016, cardiovascular diseases are responsible for 17.9 million deaths that occurred globally which accounts for 44% of NCD deaths and 31% of all global deaths(3), with over three quarters of these deaths occurring in low-and middle-income countries(2).In 2013,an estimated 1 million deaths were attributable to cardiovascular disease in sub-Saharan Africa alone, which constituted 5.5% of all global cardiovascular disease related deaths and 11.3% of all deaths in Africa(4).According to World Health Organization report on non-communicable disease, approximately 16% of all deaths in Ethiopia in 2018 were caused by cardiovascular disease (5).

The American Heart Association (AHA) categorizes the risk-factors associated with cardiovascular disease as modifiable risk factors such as smoking, dietary habits, abnormal alcohol consumption, physical activity, overweight and obesity, and non-modifiable factors such as age, gender, genetics and family history. The more risk factors an individual has, the greater the chance of developing cardiovascular disease (6).

Cardiovascular diseases can present gradually or with sudden events like acute coronary events (heart attacks), hypertensive crisis and cerebro-vascular events (strokes)that often are fatal before medical care can be given(7). Therefore, to reduce the chance of getting Cardiovascular disease in those who are at high risk of cardiovascular disease and to reduce clinical events and premature death in people with established cardiovascular disease, modification of Cardiovascular disease risk factors at varying clinical points on the cardiovascular disease continuum is very important(7).

Cardiovascular disease preventive behaviors involve taking proactive measures to slow disease progression and decrease the incidence of adverse cardiovascular events through primary, secondary and tertiary prevention mechanisms. The type of prevention intervention to be applied is decided by identifying where the individual is in the natural history of the course of cardiovascular disease (8). Primary prevention of cardiovascular disease takes place before there are precursory signs of cardiovascular disease, prior to the onset of biological risk factors or at pre-pathogenesis. Secondary prevention occurs at the stage of pathogenesis where the initial appearance of cardiovascular disease risk factors takes place. Tertiary prevention of cardiovascular disease involves prevention of disease progression and reducing limitations and disability that may result from cardiovascular disease.

The best practices for prevention and reduction of cardiovascular disease involve assessing the perception of respondents about their risk and their engagement in protective behaviors. Since bank workers spend most of their times working in offices, they are at a greater risk of contracting cardiovascular disease. Therefore, this study tries to assess bank worker’s perception about their cardiovascular disease risk and practices focused on prevention of cardiovascular disease

## Statement of the problem

Cardiovascular disease (CVD) is responsible for a considerable proportion of the mortality and morbidity of the general population. According to the World Health Organization’s prediction, Cardiovascular disease will cause 25 million deaths in 2020(9). Cardiovascular disease imposes enormous health and socio-economic burden on society and affects people when they are most efficient socially and economically(10).

Ethiopia is one of the sub-Saharan countries affected by a growing burden of non-communicable diseases (NCDs) along with pre-existing communicable diseases(11). According to a recent estimate by the World Health Organization (WHO), cardiovascular disease accounts for 16% of deaths in Ethiopia(5). Different studies conducted in Ethiopia reported an increasing burden of cardiovascular disease. A study conducted in two Hospitals of Harar town revealed an estimated prevalence of 7.2% for cardiovascular diseases(12). Another facility based study in SNNPR reported 18.8% prevalence of cardiovascular diseases(13).

Driving much of the increase in cardiovascular disease are common, preventable behavioral risk factors like tobacco use, harmful use of alcohol, physical inactivity, and unhealthy diet. According to STEPES survey in Ethiopia, these risk factors were more prevalent among 15 – 69 year old people(14). A study from Eastern Ethiopia shows that physical inactivity(76%), and hypertension (62.7%) were the most common cardiovascular disease risks factors among diabetic patients(12).

Cardiovascular diseases are becoming a public health problem in Ethiopia within a health care system that focus on communicable diseases. A study in Tikur Anbesa Hospital showed that cardiovascular diseases were responsible for 16% of deaths(15). In Addis Ababa, cardiovascular diseases were responsible for 25 % of all household deaths and 11 % of all hospital deaths (16). Similarly, data from Mortality Surveillance Program in Addis Abeba shows that non-communicable diseases were responsible for 51% of deaths, where cardiovascular disease is the leading cause with 24%(17).

Lifestyle modification along with other modifiable behavioral risk factors plays a key role in cardiovascular disease prevention and treatment. Primary prevention can rescue from modifiable (behavioral) risk factors contributes to 80% reduction of cardiovascular disease cases (18). WHO recommends a combination of population-wide and individual-based prevention and basic treatment strategies for successful control of cardiovascular disease (19). Consequently, implementation status of such interventions in Ethiopia is very low, as people have inaccurate perception about their cardiovascular disease risk status and low awareness about cardiovascular disease risk factors. To adopt healthy lifestyle behaviors related to diet, exercise, smoking and alcohol consumption, the general population must be aware of their cardiovascular disease risk level(7).

Studies conducted in Ethiopia mostly focus on assessing the magnitude of cardiovascular disease, which makes difficult to design an effective prevention plan. So, to design cardiovascular disease preventive strategies to workers who spend most of their time in office chairs, knowing their perception level has a great importance. Therefore, this study aims at studying the perception of bank workers to engage in cardiovascular disease preventive behaviors.

.

## Significance of the study

This study attempts to assess the perception of bank workers about cardiovascular disease and cardiovascular disease preventive behaviors, their perceived ability to engage in cardiovascular disease preventive behaviors as well as identify barriers for practicing cardiovascular disease preventive behaviors in different Banks of Hossana town. Understanding the perception of bank workers about cardiovascular diseases and their risk factors is essential in designing work place policies that help to improve worker’s health. The findings of this study could be used to reduce barriers to practice cardiovascular disease preventive behaviors, and to enhance the benefits by developing culturally appropriate communication materials to increase bank worker’s engagement in cardiovascular disease preventive practices. Moreover, the findings may also help in designing an effective intervention for workers in similar working environment. Therefore, it is important to determine factors that influence their decision to engage in cardiovascular disease preventive practices.

# Chapter two

# Literature review

## Prevalence of CVDs

Cardiovascular diseases are the number one cause of death globally; more people die annually from cardiovascular diseases than from any other cause. An estimated 17.9 million people died from cardiovascular disease in 2016, representing 31% of all global deaths. Of these deaths, 85% are due to heart attack and stroke(3).

The public health impact of cardiovascular disease is very high and increasingly affecting developing countries. The problem had initially been very rare in Africa, but recently it is increasing due to population dynamics, epidemiological transitions and/or increase in engagement of the community in cardiovascular disease risk factors. Cardiovascular disease related deaths contributed to 38% of all non-communicable disease related deaths in Africa, reflecting the growing threat of both non-communicable disease and cardiovascular disease (4).

According to World Health Organization report of 2018, African countries have a varied prevalence of cardiovascular diseases. Among north African countries, 40% of Moroccans, 44% of Tunisians, 26% of Algerians, 35% of Libyans, 36% of Cape Verde and 40% of Egyptians were among with higher rate of cardiovascular deaths(5). Among sub-Saharan African countries higher prevalence of cardiovascular deaths were observed in Sudan (28%), Djibouti (19%), Madagascar (20%), Ghana (19%), Eritrea (19%), Namibia (17%), South Africa (19%) and Seychelles (34%). Whereas, Kenya (8%), Somalia (10%), South Sudan (10%), Uganda (10%), and Zambia (10%) were sub-Saharan African countries with lower prevalence rate of cardiovascular disease death rates(5).

In the last few years, the lifestyle of the Ethiopian population has been changing due to urbanization and demographic transition(20).As a result, the burden of non-communicable diseases (NCDs) could be on the rise. World health organization recent estimate shows that non-communicable diseases are estimated to account for 39% of all deaths, where 42% of non-communicable deaths are attributable to cardiovascular diseases(5).

A retrospective study conducted in Tikur Anbesa Hospital showed that cardiovascular diseases were responsible for 16%of deaths among diabetic admissions(15), while a community based study conducted in Addis Ababa reported that cardiovascular diseases are responsible for 24% of the deaths(17). A study conducted in eastern Ethiopia among diabetic patients shows a 7.2% prevalence of cardiovascular disease(12).

According to a study conducted in Emergencies of Addis Abeba specialized hospital, cardiovascular emergency accounts for 11% of the medical emergency admission. Where majority of the patients were presented with Rheumatic valvular heart diseases (40%), followed by hypertension (26%) and ischemic heart disease (15%). The study also revealed that 25% of the patients were died during their hospital stay due to congestive heart failure (44%), stroke (22%) and Cardiac arrest (11%)(21).

In a systematic review of studies conducted in Ethiopia between 1960 and 2011, cardiovascular disease was reported as the prevalent causes of morbidity (range 4–24 %), the main causes of hospital admission, especially among those older than 60 years (range 3–31 %), the leading causes of medical intensive care unit admission (range 8.9–9.8 %) and among the major causes of mortality (range 6.5–24 %)(22). This increase in non-communicable diseases is expected for the future especially in relation to "westernization" of our diet and lifestyle changes in the urban setting of Ethiopia.

## Risk factors of CVD

Risk factors or underlying contributors to cardiovascular disease are in fact behaviors or conditions that increase the risk of the disease like sedentary lifestyle, smoking and high-fat diet and recognized as the behavioral and main risk factors for developing cardiovascular disease (6).The AHA classifies cardiovascular disease risk factors as modifiable (can be treated or controlled)and non-modifiable (cannot be treated or controlled). Non-modifiable risks include age, gender, heredity, and race. Modifiable risks include hypertension, overweight and obesity, diabetes mellitus, high low density lipoprotein, low high density lipoprotein, physical inactivity, atherogenic diet, tobacco use, consuming more than 1-2 alcoholic drinks per day, and stress. The more risk factors an individual has, the greater the chance of developing cardiovascular disease (23).

Based on CDC assessment, tobacco use is one of the main risk factor for contacting cardiovascular disease. Where cigarette smokers are 2-4 times more likely to develop cardiovascular disease than non-smokers(24). In a study conducted in Iran, 22% of cardiovascular disease occurs due to lack of physical activity, 22%from smoking, 13% due to lack of fruits and vegetables in daily diet, and 62% due to high blood pressure(25). A study conducted to examine cardiovascular disease -related health beliefs among Karen refugees resettled in the US revealed that, 25.6% of participants reported consuming a low-salt diet, 18.5% of the participants reported regular physical activity, and 19% of the participants were current smokers(26).

A descriptive study conducted to assess the relationship between health beliefs related to perceived cardiovascular disease (CVD) severity and health promoting behaviors among female registered nurses revealed that marital status, average sleep hours per day, and total household income had a statistically significant association with cardiovascular health promotion behaviors(27).

A study conducted in eastern Ethiopia among diabetic patients revealed that the rate of physical inactivity is 76%, smoking habit was reported in 7% of participants, and family history of CVD was found among 8.2% of participants(12).Another study conducted in Jimma, Ethiopia found that hypertension, obesity, physical inactivity(55.1%), and dyslipidemia were common CVD risk factors among diabetes patients(28).

Most of the cardiovascular disease risk was attributable to lifestyle and behavioral patterns, and these behaviors can be modified given the right sensitization and education strategies. However, some risk factors, such as age, ethnicity, and gender, obviously cannot be modified. The hazards of alcohol use, smoking, high blood pressure, high cholesterol, and overweight/obesity are globally widespread and have large health effects(29).

**Health belief model**

Behavioral and social science theories offer a framework for understanding the rationale for why people participate in health-protecting, health-risking, and health compromising activities. To that end, theory development and application are useful for understanding factors that influence the adoption or maintenance of cardiovascular disease preventive health behaviors, especially when used to plan, implement, and evaluate health promotion programs(30).

Health belief model is one of the oldest theoretical model and widely used in health behaviour research. It is used by researchers to explain change and maintenance of health-related behaviors and as a guiding framework for health behavior interventions. The HBM contains several primary concepts that predict why people will take action to prevent, to screen for, or to control illness conditions; these include perceived susceptibility, seriousness, benefits and barriers to a behavior, cues to action, and self-efficacy(31,32).

Perceived susceptibility refers to the subjective opinion of the risk of contracting a condition. Whereas, perceived severity is the subjective opinion of the seriousness of a condition and its consequences(32). Individuals have different opinions regarding their susceptibility and seriousness of the condition. A study conducted among female registered nurses shows that perceived susceptibility and severity were associated the likelihood of taking part in CVD preventive behaviors(27).

Perceived benefits refers to the subjective opinion of the effectiveness of a behavior toward decreasing a disease threat(30). When personal susceptibility to a condition is accepted by the individual and there is a move toward adopting health protective behaviors, the behaviors taken will be influenced by beliefs concerning the effectiveness of adopting the behaviors. An individual who has beliefs about high personal susceptibility and high severity would not be likely to accept any recommended health actions unless the actions were believed to be effective for decreasing the health threat(33).

Perceived barriers are the subjective opinions of the tangible and psychological expenditures related to participating in the advised action(30). Although the belief may exist that a given action may have effectiveness in decreasing the seriousness of a disease, the individual may simultaneously view the action as painful, upsetting, expensive, or inconvenient(33).

Cues to action are factors that activate readiness to take the advised action. They are the instigating events that set the movement toward performing the advised action in motion(30). Cues may be internal or external, and the intensity of the triggering cue varies by perceived susceptibility and severity(33).

Self-efficacy is the conviction that the advised behavior can be successfully executed(30). The individual must have beliefs of both competences to perform the behavior and confidence that they can triumph over the perceived barriers and achieve success.

A research conducted by review of literature of different studies that used the Health Belief Model, identified that 86% of the studies reviewed supported perceived susceptibility as a positive predictor of preventive health behaviors(32). Whereas, a cross-sectional study conducted on 180 women of child bearing age, self-efficacy, level of education and perceived susceptibility were stronger predictors for practicing cardiovascular disease preventive behavior(34).

A cross-sectional study conducted on 112 individuals who are at risk of cardiovascular disease in Birjand city of Iran in 2016 revealed that knowledge, perceived barriers, perceived benefits, and perceived self-efficacy have correlated with individuals’ adoption of preventive behaviors against cardiovascular disease(35). Similarly, another study conducted in Iran on patients at risk of cardiovascular diseases showed that occupation, physical activity, type and frequency of physical activity, smoking and awareness of cardiovascular diseases affect the respondents’ perception of barriers and benefits of adopting certain behaviors for the prevention of cardiovascular diseases (36)

## Conceptual framework

**Socio-demographic variables**

- Age
- Sex
- Marital status
- Educational status
- Experience
- Knowledge about CVD

**Likelihood of taking part in CVD preventive behaviour**

**Cues to action**

- - Media information
  - Health professional’s advice
  - Friends and family support
  - Family history of CVD

**Modifying factors**

- **Perceived susceptibility**
  - Risk of getting CVD
- **Perceived Severity**
  - Seriousness of CVD

**Perceived Benefit**

- - Decrease risk of CVD

**Perceived Barriers**

- - Decrease risk of CVD

**Perceived Self-efficacy**

**Perceived Threat of CVD**

**Perception about preventive behaviour**

Figure 1. Conceptual framework of the study by using health belief model

# Chapter three

# Objective

## General objective

Is to assess the perception of Bank workers towards cardiovascular disease prevention behaviors in Hossana town, South Ethiopia

## Specific Objectives

- To assess the perception of bank workers towards cardiovascular disease preventive behavior
- To assess factors associated with cardiovascular disease preventive practices of bank workers

# Chapter four

# Method and Material

## Study area and period

The study will be conducted from February 11/2020 to April 20/2020, in Hossana town. Hossana town is the capital of Hadiya Zone, which is located 232km from Addis Abeba, the capital of Ethiopia, and 194km from regional city Hawassa.

In Hossana town there are 16 Banks with 39 branches (including both Governmental and Private Banks). All the banks have a total of 681 workers.

Regarding health facilities, there is one Referral Hospital, 3 health centers, and 2 pharmacies,1 surgical specialty center, 27 medium clinic, 18 first level clinic, 39 drug stores and 1 public diagnostic laboratory 3 whole sell are available in Hossana town.

## Study design

A cross sectional study will be employed to assess respondents’ perception about CVD and CVD preventive behaviors.

## Population

### *Source population*

All bank workers who are currently working in Hosanna town will be used as a source population.

### *Study population*

The study population will be sampled bank workers who are currently working in Hosanna town.

## Eligibility criteria

### *Inclusion criteria*

- Respondents who are currently working and available during data collection time in different banks in Hossana town will be included in the study.

### *Exclusion criteria*

- 1. Respondents who are sick and transferred from branches during the time of data collection

4.6 Sample size determination and sampling procedure

### *Sample size*

The sample size will be determined by using single population proportion formula considering the following parameters;

Since there is no study conducted on bank workers perception about cardiovascular disease preventive behaviors, sample size is calculated by assuming that 50% of the workers will be engaged in cardiovascular disease preventive behaviors

P= 50%

$Z_{\left( 1-\frac{\alpha}{2} \right)}$= Z-score at 95% confidence interval = 1.96

d= Acceptable margin of error (precision of measurement) = 5%

The Possible Non-response rate=5%

The formula for calculating the sample size (n) is:

$$n=\frac{\left( Z_{\left( \frac{\alpha}{2} \right)} \right)^{2}P\left( 1-P \right)}{d^{2}}$$

$$n=\frac{\left( 1.96 \right)^{2}0.5\left( 0.5 \right)}{{(0.5)}^{2}}=384$$

Since the total study population is below 10,000, the final sample size will be determined by using a correction formula. Currently there are 681 workers were working in 39 branches in the town.

$n_{final}=\frac{n}{1+\frac{n}{N}}=\frac{384}{1+\frac{384}{681}}=$246

Final sample size by considering a 5% possible non-response rate, it becomes 258.

### *Sampling procedure*

In Hossana town there are 16 banks and these banks have a total of 39 branches. Then the number of staffs in each bank will be known and the sample size will be distributed to all banks proportionate to the number of staffs in each bank. The final respondents in each bank will be selected by simple random sampling method by using a sampling frame of workers in each bank.

**Banks in Hosanna town**

**Commercial ban 245**

**258 Bank workers**

**Dashen Bank 40**

**Awash bank 34**

**Hibret bank 22**

PPS

**Nib Bank 70**

**Wogagen Bank 25**

**DebubGlobal 27**

**Birhan Bank 56**

**OromiaHibret Bank 17**

**Abesinya Bank 31**

**AnbesaBank 35**

**Adis Bank 16**

**AbayBank 15**

**Buna Bank 21**

**Lemat Bank 11**

**OromiaInt Bank 16**

**8**

**4**

**6**

**12**

**6**

**21**

**11**

**27**

**9**

**93**

**15**

**14**

**8**

**6**

**5**

**13**

Figure 2. Schematic presentation of sampling procedure to select study participants

## Data collection and measurement

### *Data collection instrument*

Data will be collected by using a self-administered structured questionnaire adopted from various studies conducted by using a health belief model. The questionnaire is developed in English and will be translated into Amharic, then back translated to English by person blind to the original English version to facilitate reliable responses and to keep the original meaning of the instrument.

The questionnaire consists of the following parts;

**Socio-demographic characteristics**: - includes age, sex, educational level, marital status, monthly income, position in the bank, and work experience.

**Knowledge:** - respondents’ knowledge about risk factors and prevention methods of cardiovascular disease will be assessed by 7 questions.

**Behavioral factors:** respondents’ practice of cardiovascular disease behavioral risk factors will be assessed by 12 questions.

**Health Belief model constructs:** -respondents’ perception about cardiovascular disease will be assessed by using HBM constructs; perceived susceptibility (4 items), perceived severity (7 items), perceived benefits (5 items), perceived barriers (9 items), perceived self-efficacy (6 items), cues to action (4 items) and practice of cardiovascular disease preventive behaviors (5 items). Each items will be measured by five-point Likert scale ranging from (1) strongly disagree to (5) strongly agree. Negatively worded questions will be reverse scored (How?). The score of all items will be summed and higher score reflect higher perception about cardiovascular disease and cardiovascular disease preventive measures.

### *Pre testing of the questioner*

A pre-test of the questionnaire will be done using 5% of the sample size of the study in banks where study participants were not selected to validate the appropriateness of the tool, check for clarity of the questions and to eliminate ambiguity, difficult wordings or unacceptable questions. At the end of the pre-testing, the average time to complete the questionnaire will also be determined.

### *Data collection methods and collectors*

The data will be collected by using self-administered structured questionnaire. Five experienced and trained data collectors will be recruited for facilitating data collection and two supervisors will supervise the data collection process. One-day training will be given for both data collectors and supervisors by the principal investigator on contents of the tool and the mechanism to collect quality and relevant data before the data collection.

## Study variables

***Dependent variable***

- Perception towards cardiovascular disease preventive behaviors

***Independent variables***

- Socio demographic Variables: Age, Sex, Educational level, Marital status, Monthly income, Work experience, knowledge about cardiovascular disease, source of information
- Family history of cardiovascular disease
- Perceived susceptibility to cardiovascular disease
- Perceived severity of cardiovascular disease
- Perceived benefits of practicing cardiovascular disease preventive behaviors
- Perceived barriers to practicing cardiovascular disease preventive behaviors
- Self-efficacy to practicing cardiovascular disease preventive behaviors
- Cues to action

## Data processing and analysis

First data will be checked manually for completeness and coded, entered and analyzed by using SPSS version 20. Descriptive statistics, bivariate logistic regressions, ANOVA, general linear and multivariate analysis will be employed and the result will be described in words and figures. P-value less than 0.05 and 95% CI will be used to declare association between independent and the dependent variables.

## Data quality control

Questionnaire will be prepared in English, translated in to Amharic and back translated to English in order to check consistency of the two versions. The tools will also be pre-tested by using 5% of the sample size.

Study participants will also be informed about the importance of the data before completing the questionnaire. Finally, the filled questionnaire will be checked for completeness and consistency of responses.

## Operational and definitions of terms and concepts

**Cardiovascular disease** – refers to a group of disorders of the heart and blood vessels like coronary heart disease (CHD), hypertension, cerebrovascular disease, peripheral artery disease, heart failure, rheumatic heart disease, and congenital heart disease.

**Knowledge about** cardiovascular disease – Refers to the knowledge of respondents about risk factors and prevention methods of cardiovascular disease. The knowledge score will be summed and divided into two levels which are good knowledge and poor knowledge using the mean knowledge score as the cutoff point.

Cardiovascular disease **preventive behaviors** - refers to behaviors practiced to prevent the occurrence of cardiovascular disease include regular physical activity, healthy food choices, and avoidance of tobacco. It will be assessed by (4) questions and the responses will be summed up and the higher score indicates respondents’ participation in cardiovascular disease preventive behaviors.

**Perceived susceptibility -** refers to respondent’s belief regarding their chance of getting cardiovascular disease. It will be assessed by 5 questions and the responses will be summed up and the higher score indicates having a high perceived susceptibility towards cardiovascular disease.

**Perceived severity -** refers to respondent’s subjective opinion of the seriousness and consequences of CVD. It will be assessed by 8 questions and the responses will be summed up and the higher score indicates having a high perceived severity about CVD.

**Perceived benefit -** refers to respondent’s perception of the effectiveness of a preventive behavior in decreasing of the threat. It will be assessed by 8 questions and the responses will be summed up and the higher score indicates having a high perceived benefit about CVD preventive measures.

**Perceived barriers -** refers to respondent’s subjective opinions of the tangible and psychological obstacles to engage in CVD preventive behaviors. It will be assessed by (9)questions and the responses will be summed up and a lower score indicates having a low perceived barrier to practice CVD preventive behaviors.

**Perceived self-efficacy -** refers to respondents’ perception on their ability to practice the recommended CVD preventive behaviors. It will be assessed by 4 questions and the responses will be summed up and the higher score indicates having a high perceived efficacy to practice the recommended CVD preventive behaviors.

**Cues to action -** refers to the factors that activate readiness to engage in CVD preventive behaviors. It will be assessed by 4 questions and the responses will be summed up and the higher score indicates a supportive cue to action to practice CVD preventive behaviors.

## Ethical consideration

The study will be conducted after securing ethical approval from Wachemo University depending on the guideline of the university. Permission will be sought from the respective banks where the study will be conducted. Finally, after informing the participants about the purpose of study, benefit and risk associated with study, written consent will be sought from each study participants before collecting the data. The participants will also be informed that their response will be kept confidential and their name will not be mentioned.

## Dissemination plan

The result of this thesis will be presented to Wachemo university college of Public Health and medical science. The study findings will also be communicated to Banks in Hossana Town and those who deserve the results. Finally, effort will be made to publish in peer-reviewed journals.

# Chapter five

# Work plan of the study

Table 1: Work plan to study the perception of bank workers in Hossana town, South Ethiopia

| No | Activities | Responsibility | Months of accomplishment | | | | | | | | | |  |
| --- | --- | --- | --- | --- | --- | --- | --- | --- | --- | --- | --- | --- | --- |
|  |  |  | Sep | Nov | Oct | Nov | Dec | Jan | Feb | March | April | May | June |
| 1 | Preparation of Thesis proposal |  |  |  |  |  |  |  |  |  |  |  |  |
| 2 | Preparation of data collection tools |  |  |  |  |  |  |  |  |  |  |  |  |
| 3 | Approval of Ethical clearance and budget securing |  |  |  |  |  |  |  |  |  |  |  |  |
| 4 | Recruitment of data collectors and data collection |  |  |  |  |  |  |  |  |  |  |  |  |
| 5 | Data coding entry and Cleaning |  |  |  |  |  |  |  |  |  |  |  |  |
| 6 | Data analysis |  |  |  |  |  |  |  |  |  |  |  |  |
| 7 | Preparation and submission of first draft |  |  |  |  |  |  |  |  |  |  |  |  |
| 8 | Second draft submission |  |  |  |  |  |  |  |  |  |  |  |  |
| 9 | Preparation of final report |  |  |  |  |  |  |  |  |  |  |  |  |
| 10 | Thesis defense |  |  |  |  |  |  |  |  |  |  |  |  |

# Chapter six

# Budget Plan

Table 2: The total budget break dawn required to study the perception of bank workers in Hossana town, South Ethiopia

| **S. No** | **Budget category** | | **Participants** | **No of participants** | **No of days** | **Unit Cost** | **Total cost** | | **Remark** |
| --- | --- | --- | --- | --- | --- | --- | --- | --- | --- |
|  |  |  |  |  |  |  | **Birr** | **Cent** |  |
|  | **Personal cost** | *Training* | *Data collectors* | 5 | 1 | *100* | *500* |  |  |
|  |  |  | *Supervisors* | 2 | 1 | *100* | *200* |  |  |
|  |  | *Data Collection* | *Data collectors* | 5 | 20 | *100* | *10,000* |  |  |
|  |  |  | *Supervisors* | 2 | 20 | *100* | *4,000* |  |  |
|  |  | *Mobile card* | *PI, DC, Sup* | 8 | 1 | *300* | *2,400* |  |  |
|  |  | ***Subtotal cost*** | | | | | ***17,100*** |  |  |
|  | **Stationery cost** | **Item** | **Unit** | **Quantity** | | **Unit price** |  |  |  |
|  |  | *Paper* | *Ream* | *2* | | *120* | *240* |  |  |
|  |  | *Pencil* | *Piece* | *16* | | *2.50* | *40* |  |  |
|  |  | *Pen* | *Piece* | *16* | | *5* | *80* |  |  |
|  |  | *Sharper* | *Piece* | *8* | | *2.50* | *20* |  |  |
|  |  | *Flash Disk* | *Piece* | *2* | | *300* | *600* |  |  |
|  |  | *Notebook* | *Piece* | *8* | | *30* | *240* |  |  |
|  |  | *Proposal Print* | *Paper* | *40*3* | | *3* | *360* |  |  |
|  |  | *Thesis print* | *Paper* | *60*3* | | *3* | *540* |  |  |
|  |  | *Questionnaire* | *Paper* | *258*5* | | *2* | *3870* |  |  |
|  |  | ***Subtotal cots*** | | | |  | ***5,990*** |  |  |
| **Grand Total** | | | | | | | **23,090** |  |  |

# Refferences

1. World Health Organization. Cardiovascular Disease [Internet]. 2009. Available from: http://www.who.int/mediacentre/factsheets/fs317/en/index.html

2. World Health Organization. WHO fact sheet on cardiovascular diseases. 2017; Available from: https://www.who.int/news-room/fact-sheets/detail/cardiovascular-diseases-(cvds)

3. World Health Organization. Global Health Estimates 2016: Deaths by Cause, Age, Sex, by Country and by Region, 2000–2016. Geneva. 2016;

4. Mensah GA et al. Mortality from cardiovascular diseases in sub‑Saharan Africa, 1990‑2013. 2013;

5. World Health Organization. Noncommunicable diseases country profiles 2018. Geneva. 2018.

6. American Heart Association; Heart disease and stroke Statistics-2009. 2009.

7. Dagnaw WW, Yadeta D, Feleke Y, Kebede T. Ethiopian National Guideline on Clinical and Programmatic Management of Major Non Communicable Diseases. 2016;

8. Bairey, M.C. N, Alberts, M. J., Balady, G. J., Ballantyne, C. M., Berra, K., Black HR, Blumenthal RSD. A report of the American College of Cardiology Foundation/American Heart Association/American College of Physicians Task Force on Competence and Training on prevention of cardiovascular disease. Journal of the American College of Cardiology. 2009;54:1336–63.

9. World Health Organization. International Cardiovascular Disease Statistics [Internet]. Available from: http://www.sld.cu/galerias/pdf/servicios/hta/international_cardiovascular_disease_statistics.pdf

10. Baghianimoghadam MH, Mirzaei M RT. Role of Health Beliefs in Preventive Behaviors of Individuals at Risk of Cardiovascular Diseases. Health System Research. 2012;8(7):1151–8.

11. Tesfaye F. Epidemiology of Cardiovascular Disease Risk Factors in Ethiopia: The rural–urban gradient, in Epidemiology and Public Health Sciences. Umeå University Department of Public Health and Clinical Medicine. 2008;87.

12. Abdosh T, Weldegebreal F. Cardiovascular diseases risk factors among adult diabetic patients in eastern Ethiopia. 2019;

13. Endriyas M, Mekonnen E, Dana T, Daka K, Misganaw T, Ayele S, et al. Burden of NCDs in SNNP region , Ethiopia : a retrospective study. 2018;18(520).

14. Ethiopian Public Health Institute. Ethiopia STEPS report on rik factors for Non-communicale diseaes and prevalence of selected NCDs. 2016;

15. Seyoum B, Abdulkadir J, Gebregziabiher F et al. Analysis of diabetic patients admitted to Tikur-Anbessa Hospital over eight years. Ethiopian Journal of Health Dev. 1999;13:9–13.

16. Misganaw A, Mariam DH, Ali A, Araya T. Epidemiology of Major Non-communicable Diseases in Ethiopia : A Systematic Review Search strategy. 2014;32(1).

17. Misganaw A, Mariam DH AT. The Double Mortality Burden Among Adults in Addis Ababa , Ethiopia , 2006-2009. Preventing Chronic Disease. 2016;

18. Shamas M., Samavat T. Prevention and control of cardiovascular disease. 2012.

19. World Health Organization. Prevention of Cardiovascular Disease: Guideline for assessment and management of cardiovascular risk. 2007; Available from: https://www.who.int/cardiovascular_diseases/guidelines/Full text.pdf

20. Population FD of E, Census Commission. Summary and Statistical report of the 2007 population and housing census. Addis Ababa: Central Statistics Authority. 2008;

21. Tsegalem Hailemariam. Prevalence of Cardiovascular Emergencies in Specialized Hospital, Addis Ababa. Open access. 2014;4(4):8–12.

22. Misganaw A, Mariam DH, Ali A, Araya T. Epidemiology of Major Non-communicable Diseases in Ethiopia : A Systematic Review Search strategy. 2014;32(1):1–13.

23. American Heart Association. Fact Sheet on Cardiovascular Disease. 2008; Available from: http://www.americanheart.org/downloadable/heart/1203961239113CDC Fact%0A Sheet 2-15-08 final.pdf

24. CDC Fact sheet. Health Effects of Cigarette Smoking. 2008;

25. Pourreza B. Effect of Education Programs on Preschool Children, Their Teachers, Principals and Parents Cardiovas Health. Journal of Guilan University Medical Sciences. 2013;22(85):67–79.

26. Kamimura A, Sin K, Pye M, Meng H. Cardiovascular Disease – related Health Beliefs and Lifestyle Issues Among Karen Refugees Resettled in the United States From the Thai-Myanmar ( Burma ) Border. Journal of Preventive Medicine & Public Health. 2017;50:386–92.

27. Deborah Mcclendon. Perceived Susceptibility of Cardiovascular Disease as a Moderator of Relationships between Perceived Severity and Cardiovascular Health Promoting Behaviors among Female Registered Nurses. Georgia State University [Internet]. 2011; Available from: https://scholarworks.gsu.edu/nursing_diss/22

28. Tamiru S and Alemseged F. Risk factors for cardiovascular diseases among diabetic patients in southwest Ethiopia. Ethiopian Journal of Health Sciences. 2010;20:121–8.

29. Lopez AD, Mathers CD, Ezzati M et al. Global and regional burden of disease and risk factors, 2001: systematic analysis of population health data. Lancet. 2006;367:1747–1757.

30. Diclemente RJ, Crosby RA, Kegler MC. Emerging Theories in Health Promotion Practice and Research: Strategies for Improving Public Health. John Wiley & Sons Inc.; 2002.

31. Karen Glanz BK, Rimer and KV. Health behavior and health education : theory, research, and practice. 4th ed. San Francisco: Published by Jossey-Bass; 2008.

32. Janz, N. K. & Becker MH. The Health Belief Model: A decade later. Health Education Quarterly. 1994;11(1):1–47.

33. Irwin M. Rosenstock. Historical origins of the health belief model. Charles B.Slack inc. 1974.

34. Mohtasham Ghaffari, Sakineh Rakhshanderou, Ali Safari-Moradabadi ZA. Correlates of Cardiovascular Diseases-related Nutritional a Research from Iran. Middle east journal of family medicine. 2018;16(2).

35. Sharifzadeh G, Moodi M, Majd HM, Musaee I. Application of Health Belief Model in predicting preventive behaviors against cardiovascular disease in individuals at risk. 2017;1(2):64–9.

36. Tol Azar, Esmaeili Shahmirzadi Sima, Shojaeizadeh Davoud, Eshraghian Mohamad A, Mohebbi Bahram. Determination Of Perceived Barriers And Benefits Of Adopting Health-Promoting Behaviors in Cardiovascular Diseases Prevention: Application Of Preventative Behavior Model. 2012;204–14.

#

# Annex: English version questionnaire

**PERCEPTION OF BANK WORKERS TOWARDS CARDIOVASCULAR DISEASE PREVENTION IN HOSSANA TOWN: APPLICATION OF HEALTH BELIEF MODEL**

**Consent Form**

Dear Sir/Madam,

Good morning/Good afternoon, my name is______________. I am working as a data collector for a research study on the perception of Bank workers towards cardiovascular disease preventive behaviors in Hossana town.

The purpose of this study is to assess the perception of Bank workers towards cardiovascular disease preventive behaviors and associated factors in Hossana town, and findings from this study can provide useful input to different program managers who are working on preventing cardiovascular disease. As a Bank worker you are playing a great role in the socio-economic field of the country. The reason we select you to participate in this study is to get good information regarding your perception towards cardiovascular disease preventive behaviours and use it to design programs to improve the health of bank workers in different area. Your participation in the study is voluntary and you have full right either to participate in the study or decline to participate at all. By participating in this study, you will not get direct benefit, but your honest response will have a useful input in the prevention of cardiovascular diseases among bank workers and the general population. Your participation in this study will not have any risk or harm, except it takes 20 or 25 minutes of your time and we greatly appreciate your cooperation. The information that we get from you is completely confidential and you don’t need to mention your name. The data will not be used for other purpose other than achieving the objective of the study.

If you have any question which is not clear for you, you are welcomed to ask at any time. If you need any further information or explanation regarding to this study, you can contact the principal investigator Mrs Lemlem K/Yesus; Phone number_+251911817145; Email address  [lemlemkyesus@gmail.com](mailto:%20lemlemkyesus@gmail.com).

Do I have your permission to continue? Yes No

If yes, thank and proceed to completing the questionnaire.

If no, thank and go to the next participan

| **General Information** | Date _____________  Respondent ID ______________  Company/Bank name _______________ |
| --- | --- |

**Part I: Socio demographic information**

**Instruction: *for each of the following questions please circle the number of alternative(s) that fit for your response.***

| **No** | **Question** | **Response** | **Skip** |
| --- | --- | --- | --- |
| 101 | Age in years | _______years |  |
| 102 | Sex | 1. Male 2. Female |  |
| 102 | Educational level | 1. Only read and write 2. High school complete 3. Diploma 4. Degree 5. Masters |  |
| 103 | Marital status | 1. Single 2. Married 3. Divorced 4. Widowed |  |
| 104 | Religion | 1. Orthodox 2. Protestant 3. Muslim 4. Catholic 5. Others |  |
| 105 | Work experience in years | _______years |  |
| 106 | Current position/level in the bank | 1. Beginner 2. Officer position 3. Mid-level manager 4. Higher level manager |  |
| 107 | How many hours you work (per week) |  |  |
| 108 | Monthly income in ETB | ___________Birr |  |

**Part 2: Knowledge about CVD**

| **No** | **Question** | **Response** | **Skip** |
| --- | --- | --- | --- |
| 201 | Do you know about CVDs? | 1.Yes  2. No |  |
| 202 | If yes, which type of CVD do you know? List all you know | 1. Coronary heart disease (CHD) 2. Hypertension 3. Stroke 4. Heart failure 5. Rheumatic heart disease 6. congenital heart disease 7. Others (Specify______) |  |
| 203 | From where do you hear about CVD? (Source of information) (more than one response is possible) | 1. Mass media 2. Social media 3. Health workers 4. From work place 5. From friends 6. Others(Specify_____) |  |
| 204 | Do you know the risk factors for CVD? | 1.Yes  2. No |  |
| 205 | If yes, can you mention risk factors for CVD? (more than one response is possible) | 1. Lack of physical activity 2. Excessive alcohol intake 3. Tobacco Smoking 4. Reducing excess intake of salt and fat in daily diet 5. Staying long hours on office chairs 6. Passive smoking 7. Others(Specify_____) |  |
| 206 | Do you know how CVD can be prevented? | 1.Yes  2. No |  |
| 207 | If yes, can you mention some of the preventive measures of CVD? (more than one response is possible) | 1. Physical activity 2. Reduce alcohol intake 3. Quit tobacco Smoking 4. Avoidance of passive smoking 5. Consuming more fruit, nuts, seeds, vegetables 6. Reducing salt and fat from daily diet 7. Others(Specify_____) |  |

**Part 3: Behavioral risk factors**

| **No** | **Question** | **Response** | **Skip** |
| --- | --- | --- | --- |
| 301 | Have you ever smoked cigarette? | 1.Yes  2. No |  |
| 302 | Do you currently smoke cigarette? | 1.Yes  2. No |  |
| 303 | For how long did you smoke? | _________years |  |
| 304 | Have you exposed to passive smoking at home, workplace or other areas? | 1.Yes  2. No |  |
| 305 | Do you engage in any physical activity? | 1. Yes 2. No |  |
| 306 | If yes, in which type of physical exercise do you currently engaged? | 1. Moderate physical activity   (30 minutes for 5 days/week)   1. Vigorous physical activity   (15 minutes for 5 days/week)   1. Working in gym 3 or more days/week 2. Participate in football events 3 or more days/week 3. Others (Specify_______) |  |
| 307 | If no, why you didn’t engage in physical exercise? | 1. Don’t see the benefit of physical exercise 2. Being busy with office work 3. Other (Specify_______) |  |
| 308 | Do you take alcohol? | 1.Yes  2. No |  |
| 309 | If yes, how do you describe your alcohol intake? | 1.Somitimes  2. Occasionally  3. Regularly |  |
| 310 | How much unit alcohol do you take in a day? | _________ units  One unit = half pint of beer (5 % alcohol), 100 ml of wine (11 % alcohol), 25 ml of alcohol (40% alcohol) |  |
| 311 | Do you regularly eat fruits and vegetables in your daily meal? | 1. Yes 2. No |  |
| 312 | Do you regularly consume salt and fats in your daily meal? | 1. Yes 2. No |  |
| 313 | History of CVD | 1. Yes 2. No |  |
| 314 | Family history of CVD | 1. Yes 2. No |  |

**Part 4: Perception questions**

| **No.** | **Question** | **Response** | | | | |
| --- | --- | --- | --- | --- | --- | --- |
|  | **Perceived susceptibility** | **Strongly disagree** | **Disagree** | **Neutral** | **Agree** | **Strongly agree** |
| 401 | In my opinion, as I am working in office, I am susceptible to CVD | 1 | 2 | 3 | 4 | 5 |
| 402 | I feel I will get CVD sometimes during my life | 1 | 2 | 3 | 4 | 5 |
| 403 | It is likely that I will get CVD | 1 | 2 | 3 | 4 | 5 |
| 404 | I worry a lot about having CVDs | 1 | 2 | 3 | 4 | 5 |
|  |  |  |  |  |  |  |
|  | **Perceived severity** | **Strongly disagree** | **Disagree** | **Neutral** | **Agree** | **Strongly agree** |
| 405 | The thought of CVD scares me | 1 | 2 | 3 | 4 | 5 |
| 406 | When I think about CVD my heart beats faster | 1 | 2 | 3 | 4 | 5 |
| 407 | CVD would affect my work | 1 | 2 | 3 | 4 | 5 |
| 408 | I am afraid to think about CVD | 1 | 2 | 3 | 4 | 5 |
| 409 | If I got CVD, it would be more serious than other disease | 1 | 2 | 3 | 4 | 5 |
| 410 | CVD is not serious as other diseases | 1 | 2 | 3 | 4 | 5 |
| 411 | Death resulting from CVD is rare | 1 | 2 | 3 | 4 | 5 |
| ­­­­­­ |  |  |  |  |  |  |
|  | **Perceived benefits** | **Strongly disagree** | **Disagree** | **Neutral** | **Agree** | **Strongly agree** |
| 412 | When I do physical exercise, I am doing something to take care of myself | 1 | 2 | 3 | 4 | 5 |
| 413 | Regularly screening for CVD may help me to take care of myself | 1 | 2 | 3 | 4 | 5 |
| 414 | Regular physical exercise decreases the risk of CVD | 1 | 2 | 3 | 4 | 5 |
| 415 | If I do physical exercise regularly, I don’t worry much about CVD | 1 | 2 | 3 | 4 | 5 |
| 416 | If I do physical exercises regularly, I will decrease my chances of exposure to CVD | 1 | 2 | 3 | 4 | 5 |
| ­­­ |  |  |  |  |  |  |
|  | **Perceived barriers** | **Strongly disagree** | **Disagree** | **Neutral** | **Agree** | **Strongly agree** |
| 417 | Doing physical exercise is difficult to me | 1 | 2 | 3 | 4 | 5 |
| 418 | Doing physical exercise will take too much of my working hour | 1 | 2 | 3 | 4 | 5 |
| 419 | It’s hard to remember to do physical exercise regularly | 1 | 2 | 3 | 4 | 5 |
| 420 | Physical exercise is not necessary if I have regular screening for CVD | 1 | 2 | 3 | 4 | 5 |
| 421 | It’s difficult for me to do regular physical exercises | 1 | 2 | 3 | 4 | 5 |
| 422 | I have other problems more important than doing physical exercise | 1 | 2 | 3 | 4 | 5 |
| 423 | If I engage in regular physical exercise, it would lead me to worry about CVD | 1 | 2 | 3 | 4 | 5 |
| 424 | My friends/family would make fun of me if I engage in regular physical exercise | 1 | 2 | 3 | 4 | 5 |
| 425 | Doing regular physical exercise would require starting a new habit, which is difficult for me | 1 | 2 | 3 | 4 | 5 |
|  |  |  |  |  |  |  |
|  | **Perceived self-efficacy** | **Strongly disagree** | **Disagree** | **Neutral** | **Agree** | **Strongly agree** |
| 426 | I know how to do regular physical exercise to prevent CVD | 1 | 2 | 3 | 4 | 5 |
| 427 | I know when to do physical exercise if I have to do | 1 | 2 | 3 | 4 | 5 |
| 428 | I am confident I can do regular physical exercise | 1 | 2 | 3 | 4 | 5 |
| 429 | I think I can control CVD risk factors by myself | 1 | 2 | 3 | 4 | 5 |
| 430 | I can visit a health care provider to check my health status | 1 | 2 | 3 | 4 | 5 |
| 431 | I could feel any abnormalities in my health status | 1 | 2 | 3 | 4 | 5 |
|  |  |  |  |  |  |  |
|  | **Cues to action/Motivation** | **Strongly disagree** | **Disagree** | **Neutral** | **Agree** | **Strongly agree** |
| 432 | I have heard good things about physical exercise in preventing CVD | 1 | 2 | 3 | 4 | 5 |
| 433 | I have good support from my office to do regular physical exercise | 1 | 2 | 3 | 4 | 5 |
| 434 | I have seen friends engaged in regular physical exercise | 1 | 2 | 3 | 4 | 5 |
| 435 | I have seen my friends suffering from CVDs | 1 | 2 | 3 | 4 | 5 |

**Part 5: Perception about CVD preventive behaviours**

|  | **Perception about CVD preventive behaviours** | **Strongly disagree** | **Disagree** | **Neutral** | **Agree** | **Strongly agree** |
| --- | --- | --- | --- | --- | --- | --- |
| 501 | If I do regular physical exercise, I can reduce the risk of CVD | 1 | 2 | 3 | 4 | 5 |
| 502 | Refraining from tobacco smoking will reduce my risk of CVD | 1 | 2 | 3 | 4 | 5 |
| 503 | Regular taking of alcohol will increase the risk of CVD | 1 | 2 | 3 | 4 | 5 |
| 504 | Reducing salt and fat intake from daily diet will reduce the risk of CVD | 1 | 2 | 3 | 4 | 5 |
| 505 | Regular screening for possible CVD will reduce complications associated with CVD | 1 | 2 | 3 | 4 | 5 |

***Thank you for your cooperation to take part in the study!!!!***

Signature of the data collector____________________
