# Supplementary material for: Perception towards cardiovascular diseases preventive practices among bank workers in Hossana town using the health belief model
Source: PLoS One. 2022 Feb 28;17(2):e0264112. doi: 10.1371/journal.pone.0264112 (PMC8884546; doi:10.1371/journal.pone.0264112)
Supplement: Attachment — Submitted filename: Response to Reviewers.docx [file pone.0264112.s003.docx]

**Dear Academic Editor and Reviewers,**

Thank you so much for your valuable comments and interest in the publication of the manuscript. Your comments have improved the quality of our manuscript and almost all the comments are incorporated into the current manuscript. The “Revised Manuscript with Track Changes” will show how much we improved our manuscript for grammar.

**Response to Dear Academic Editor,**

Thank you so much for your valuable comments and interest in the publication of the manuscript. Your comments regarding the naming of each file and submitting them with the required name are incorporated into the current manuscript. Guidelines for resubmitting each file are considered as per your comments. The title is improved as “**Perception towards Cardiovascular Diseases Preventive Practices among Bank Workers in Hossana Town using the Health Belief Model.” In general, each and every comments of academic editor is considered in this current manuscript to improve the quality of the paper. We also thank the academic editor for this valuable comments and refining the manuscript more.**

**Response to Reviewer 1,**

Thank you so much for your insightful comments and interest in the manuscript's publication. Your suggestions improved the quality of our manuscript, and all of them have been incorporated into the current version. The points on which you require clarification were addressed one by one.

**Comment 1.** Figure 1: Message in circular box seems to be incomplete. Please correct.

**Answer:** Thank you for the comment. We improved and corrected in the current manuscript.
**Comment 2.** “Since there is no study to estimate k, it is taken as 0.25” – please clarify for better understanding.

**Answer:** We thank you for your valuable comment and clarified as it is not necessary to state in such a way. It is omitted and rephrased in better way.

**Comment 3**. Figure 2: Location of PPS heading is inappropriate. It seems that only for the banks mentioned in the middle part were involved in PS allocation technique.

**Answer:** We thank you for the comment and amended (it was editorial error) in the current manuscript. Following **comment 9**, we deleted month and year in the current manuscript.
**Comment 4**. In table 2 title: September 2020 is written. Please correct.

**Answer:** We accepted your valuable comment and edited it in the current manuscript.
**Comment 5**. Table 4: History of CVD: 0.14 (0.04, 1.07) ** - how can it be significant, since it is covering 1?

**Answer:** Thank you for your valuable comment. Sorry, “history of CVD” is not significant in adjusted OR. We accepted and edited it in the current manuscript.
**Comment** 6. Most of the references are not written uniformly and as per norm. Please correct.

**Answer:** Thank you for your valuable comment. We corrected and updated in the current manuscript.
**Comment 7**. Output of binary logistic regression is missing. What about model fitness, statistical significance of model, variation of dependent variables that can be explained from the independent variables?

**Answer:** We accepted your valuable comment and included it in the current manuscript. Those variables significant in both binary and multivariable logistic regression were included in table 4. The model fitness was measured by the Hosmer-Lemeshow goodness of fit test for logistic regression. Totally, 80.3% of the variance in the perception of CVD preventive behavioral response was explained over the independent variables by the HBM model. The Hosmer-Lemeshow goodness of fit test for logistic regression was not significant. i.e. (p>0.05)
**Comment 8**. How independent variables were chosen in multivariable regression?

**Answer:** Thank you for your valuable comment. We clarified this in methods and materials part of the current manuscript. i.e. All explanatory variables that were associated with the outcome variable in bivariate analysis with a p-value of 0.20 or less were included in the initial logistic models to increase the candidate variables for final model prediction **(According to Vittinghoff et al (2005, p.134)).**

**Comment** 9. Better to reframe the title of the tables and figures. What is the necessity of providing year and month?

**Answer:** We rephrased and deleted month and year in the current manuscript.

**Response to Reviewer #2,**

We thank you so much for your valuable comments and interest in the publication of the manuscript. We improved our manuscript as per your comment and the language is also improved accordingly. Indeed, your comments improved the quality of our manuscript and almost all the comments are incorporated into the current manuscript. The “Revised Manuscript with Track Changes” will show how much we improved our manuscript for grammar. Points that you need clarifications were answered one by one.

**Comment 1.** Explanation of Health Belief Model was not mentioned in the introduction part. The title consists of "Health Belief Model". So it is better to include description of Health Belief Model in introduction part.

**Answer:** Thank you for your valuable comment. We accepted your comment and included the description of Health Belief Model in introduction part in the current manuscript.
**Comment 2**. What is the rationality of assuming likelihood of taking preventive measures by only considering perceived benefit minus perceived barriers?

**Answer:** Thank you for your valuable comments. The very assumption of the Health Belief Model is that people are largely rational in their thoughts and actions, and will take the best health-supporting action if they feel that it is possible to address a negative health issue. In this regard, the model not only helps to explain health-related behavior and its determinants, but it can also guide the development of interventions to influence and change health-related behavior and ultimately improve health. Actually, the risk of susceptibility and severity of the diseases lead the individual to seek a solution or to rationalize the condition as parallel. Speech on the ground (rationale), HBM based intervention is always relying on improving benefits and reducing barriers. This is called discriminative score (critical value), where the mean score of the barrier is subtracted from the mean score benefits. It is suggestive that the likelihood of taking action has a cumulative effect that ends with improving benefits and reducing barriers. I hope your previous comments to include the description of the health belief model, is very important to understand the logic of using discriminative scores to determine the likelihood of taking action.
**Comment 3**. Why a total Cronbach's alpha calculated and mentioned? Already construct wise Cronbach's value was mentioned.

**Answer:** Thank you for your valuable comment. The total Cronbach's alpha was calculated as a measure of internal consistency, that is, how closely related a set of items are as a group. It is considered to be a measure of scale reliability. In fact, construct wise calculation is enough as per your comment, and a high value for alpha does not imply that the measure is one-dimensional. We accepted and amended it in the current manuscript.

**Comment 4**. SD value of perceived severity in table 3 should be in two decimal.

**Answer:** We accepted your valuable comment and edited as two decimal places in the current manuscript.
**Comment 5**. Whether perceived self-efficacy and perceived susceptibility taken into consideration as independent predictors? It should be mentioned.

**Answer:** Thank you for your valuable comment. At the very beginning, Rosenstock's Health Belief Model (HBM) is a theoretical model concerned with health decision-making. The model attempts to explain the conditions under which a person will engage in individual health behaviors such as preventative screenings or seeking treatment for a health condition if he/she thinks as susceptible and its severity harms him/her. The confidence he has will also determine whether to engage in healthy behavior. Accepting a certain behavior as a preventive action depends on once susceptibility, severity and self-efficacy (Rosenstock, 1966). We accepted and clarified it in the introduction part of the current manuscript.
**Comment 6**. What is the rationality of taking P-value <0.20?

**Answer:** Thank you for the comment. According to The only reason to include all explanatory variables that were associated with the outcome variable in bivariate analysis with a p-value of 0.20 or less were included in the initial logistic models is to increase the candidate variables for final model prediction and to remove confiders (**(According to Vittinghoff et al (2005, p.134)**). And also **Maldonado and Greenland (1993)** suggest that potential confounders be eliminated only if p => 0.20, in order to protect against residual confounding. To briefly summarize, a crude odds ratio is just an odds ratio of one independent variable for predicting the dependent variable in the presence of confiders. The adjusted odds ratio holds other relevant variables constant and provides the odds ratio for the potential variable of interest which is adjusted for the other independent variables included in the model. We accepted and clarified it in such a way in the current manuscript.
**Comment 7.** In Discussion section, it is mentioned association between perceived benefits, perceived barriers and perceived self-efficacy with preventive practices of CVD. But nowhere in the result section, these were mentioned. Explain?

**Answer:** Thank you for your valuable comment. Self-efficacy is not significant variable. Unfortunately, the word “not” is missed. Now, it is corrected and written in meaningful way. Concerning perceived benefits and perceived barriers, when we saw compositely, both are treated as outcome variable (your previous comment to add the description of health belief model gives a clue for this. Thank you for that). However, when treated separately with CVD prevention activities, they are statistically significant. It is quite important to discuss the two perception variables since they are treated as an outcome variable and we didn’t put their odds ratio in the previous document. However, we included in the table 4 as per your comment. We really appreciate your in-depth looking of our manuscript to increase the quality of it. We fully accepted and edited in the current manuscript.

**Comment 8**. Some references are too old. Use recent references.

**Answer:** Thank you for your valuable comment. We updated them in the current manuscript. In fact, some of the references are general truth about HBM and its application and also the trend of the occurrences of the diseases might be there.
