# Supplementary material for: Perception towards cardiovascular diseases preventive practices among bank workers in Hossana town using the health belief model
Source: PLoS One. 2022 Feb 28;17(2):e0264112. doi: 10.1371/journal.pone.0264112 (PMC8884546; doi:10.1371/journal.pone.0264112)
Supplement: Attachment — Submitted filename: Response to Reviewers-1.docx [file pone.0264112.s004.docx]

**Dear Academic Editor and Reviewers,**

Thank you so much for your valuable comments and interest in the publication of the manuscript. Your comments have improved the quality of our manuscript and almost all the comments are incorporated into the current manuscript. The "Revised Manuscript with Track Changes" will show how much we improved our manuscript for grammar.

**Response to Dear Academic Editor,**

Thank you so much for your interest in the publication of the manuscript. **We revised the comments of reviewer 1. We used EndNote to revise the reviewer 1 comment about references. We will also revise at the time of proofreading/author proof, if any.**

**Response to Reviewer 1,**

We thank you so much for your comments and refining our manuscript for publication. Your comments were incorporated into the current version of the revised manuscript. Points that you need clarification on were answered one by one.

**Comment 1.** In the abstract, the study period was written as 11 to 20^th^ February, 2020, while in the main text, the method section; it was written as 11 to 30^th^ February, 2020.

**Answer:** Thank you for the comment. We improved and corrected the study period as of 11 to 30^th^ February, 2020 in both sections in the current manuscript. Sorry, this mistake occurred when we were summarizing the manuscript. Now it is corrected.

**Comment 2.** Materials and methods: "study design and populations: A cross-sectional study was conducted to assess bank workers’ perception of CVD preventive behaviors in Hossana town, Ethiopia. Need not to mention in this section.

**Answer:** We incorporated your valuable comment in the current manuscript. It is omitted and rephrased in a better way. In our opinion, mentioning study design is very important to better understanding the description and arrangement of the study. This in turn helps to guide analysis and to answer research questions.

**Comment 3.** Table 3: scores were represented in mean and SD. Did you check for distribution of the scores.

**Answer:** Yes, dear reviewer. We thank you for the comment. We checked all the assumptions, whether they met or not.

**Comment 4**. References are still not uniform. References: 13 &14 etc. (et alafter name of first author); references 20 & 21 etc: 2 or 3 authors are written.

**Answer:** Thank you for your valuable comment. We updated them in the current manuscript and **used "EndNote" to revise references as per your comment and replace them where necessary**. Now it is uniform. Thank you again.
